# Fungal extracellular vesicle-mediated regulation: from virulence factor to clinical application

**DOI:** 10.3389/fmicb.2023.1205477

**Published:** 2023-09-15

**Authors:** Jie Liu, Xiaoping Hu

**Affiliations:** Department of Dermatology, Skin Research Institute of Peking University Shenzhen Hospital, Peking University Shenzhen Hospital, Shenzhen, China

**Keywords:** IFD, EVs, immune system, diagnosis, vaccine vehicles

## Abstract

Invasive fungal disease (IFD) poses a significant threat to immunocompromised patients and remains a global challenge due to limited treatment options, high mortality and morbidity rates, and the emergence of drug-resistant strains. Despite advancements in antifungal agents and diagnostic techniques, the lack of effective vaccines, standardized diagnostic tools, and efficient antifungal drugs contributes to the ongoing impact of invasive fungal infections (IFI). Recent studies have highlighted the presence of extracellular vesicles (EVs) released by fungi carrying various components such as enzymes, lipids, nucleic acids, and virulence proteins, which play roles in both physiological and pathological processes. These fungal EVs have been shown to interact with the host immune system during the development of fungal infections whereas their functional role and potential application in patients are not yet fully understood. This review summarizes the current understanding of the biologically relevant findings regarding EV in host-pathogen interaction, and aim to describe our knowledge of the roles of EV as diagnostic tools and vaccine vehicles, offering promising prospects for the treatment of IFI patients.

## 1. Introduction

Invasive fungal disease (IFD) is a severe and life-threatening condition caused by fungal infections. Recent reports indicate that fungal infections cause over 1.5 million deaths worldwide each year (GAFFI^1^), surpassing malaria (WHO^2^) and similar to tuberculosis (WHO^3^) in terms of mortality rates. This highlights the global significance of fungal infections, with an increasing number of high-risk groups being exposed to invasive fungal infections (IFIs), including individuals with hematologic malignancies and those affected by HIV/AIDS. Unfortunately, therapeutic options for IFD are severely limited by challenges such as incomplete drug efficacy, cytotoxicity, and the emergence of drug-resistant strains (Odds, 2003). This is further exacerbated by the growing population of immunocompromised patients, including individuals with HIV infection, leukemia, or cancer undergoing immunosuppressive therapy or chemotherapy (Mossad, 2018; Rawson et al., 2021). Given the global prevalence of fungal disease and the limitations in its treatment, it is essential to research the interaction between fungi and the host immune system during the development of fungal infections and to explore novel therapeutic strategies.

Extracellular vesicle (EV), including exosome, microvesicle, and apoptotic vesicle, is a lipid-bilayer-enclosed particle that is secreted by both prokaryotic and eukaryotic cells (Lotvall et al., 2014). Exosome is nanosized vesicle (30–100 nm) and its biogenesis encompasses a series of intricate steps including endosome formation, multivesicular bodies (MVBs) generation, and cargo sorting. The initiation of exosome biogenesis arises with the budding of an endosomal vesicle from the plasma membrane. There are three distinct mechanisms underlie the formation of endocytic vesicles including clathrin-mediated endocytosis (CME), caveolin-dependent endocytosis (CDE), and clathrin- and caveolin-independent endocytosis (Mayor et al., 2014). Fusion of endocytic vesicles results in the creation of intraluminal vesicles (ILVs) within early endosomes, a process governed by ESCRT-dependent and ESCRT-independent pathways that subsequently culminate in the formation of MVBs. Exosomes take shape within these ILVs, which originate within the framework of late endosomes, an essential facet of the endocytic apparatus. The sorting of cargoes into ILVs draws upon diverse organelles including the trans-Golgi network (TGN), endoplasmic reticulum (ER), and mitochondria. MVBs exhibit the capability to fuse with lysosomes, thereby undergoing lysosomal degradation (Kwon et al., 2016). Alternatively, MVBs undertake trafficking toward the plasma membrane, where membrane fusion facilitates the extracellular release of ILVs in the form of exosomes. Unlike exosomes, microvesicles (MVs) (100 nm to 1 μm) have a direct origin from the plasma membrane and are commonly categorized as ectosomes (Heijnen et al., 1999). The process governing MV formation initiates with the development of outward buds at specific membrane sites, followed by fission and eventual liberation of the vesicle into the extracellular milieu (Ratajczak et al., 2006). Molecular rearrangements within the plasma membrane transpire at the points of MV origination, leading to membrane budding. These rearrangements encompass alterations in lipid and protein composition as well as changes in Ca^2+^ levels. The perturbed Ca^2+^ levels trigger the recruitment and activation of calcium-dependent enzymes, such as scramblase and floppase, inducing subsequent modifications in the lipid composition of the plasma membrane. Notably, one of the principal attributes of MVs is the externalization of phosphatidylserine (PS) (Barteneva et al., 2013). The generation of microvesicles requires cytoskeleton components, such as actin and microtubules, along with molecular motors (kinesins and myosins), and fusion machinery (SNAREs and tethering factors), However, the precise route of MV formation is unclear.

EVs derived from fungi have generated increased attention and emerged as a promising field in recent years. Research has revealed that fungal EVs carry a range of components, including proteins, enzymes, lipids, nucleic acids, and carbohydrates, which enable them to facilitate biofilm formation, virulence factor transportation, and modulate of the host immune response (Rutter et al., 2022). Fungi release EVs that transport enzymes and metabolites involved in the infection process, thus being considered as virulence factors. Recent years have witnessed notable advancements in fungal EV research, unveiling their potential applications as immunomodulatory agents and therapeutic strategies that actively participate in biological processes, diagnosis, and therapy. However, the interaction between fungi and the host immune system remains poorly understood, and there is limited research investigating the potential clinical applications of fungal EVs. Therefore, it is imperative to summary the immune response triggered by fungal EVs in the host to facilitate their potential role in diagnosis and therapy.

## 2. Fungal EVs and virulence factors

### 2.1. Fungi infection

**Figure 1:**
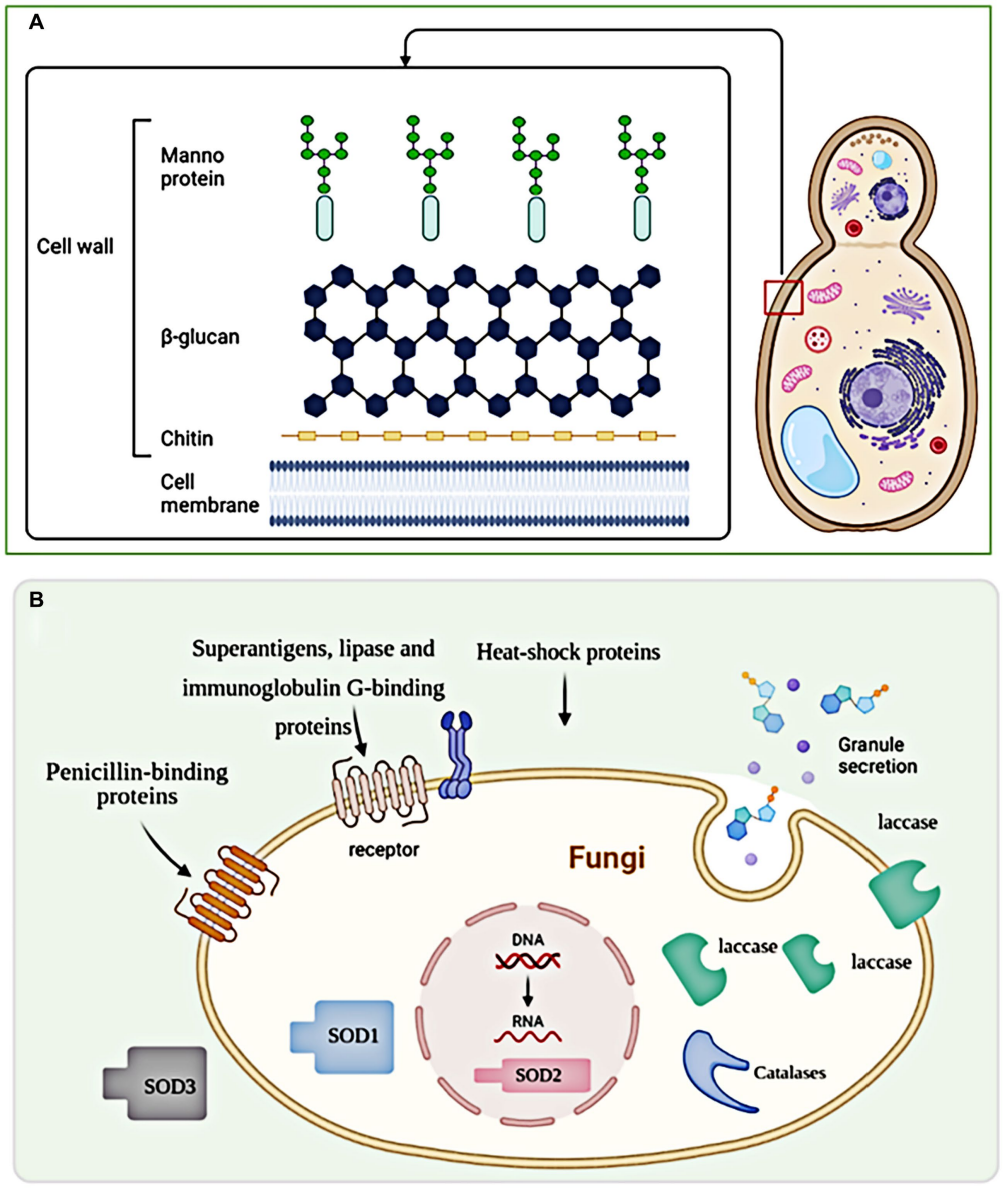
The structure of fungal cell wall and virulence factors. **(A)** The fungal cell wall is a complex structure composed of polysaccharides, proteins, and lipids that provide structural integrity and protection to fungal cells. **(B)** Exosomes, being larger vesicles, do not directly pass through the fungal cell wall but can indirectly influence it through the secretion of enzymes and other molecules involved in cell wall modification and remodeling.

Fungal infections occur through two pathways: extrinsic and intrinsic. Extrinsic infections are mainly caused by environmental fungi, while intrinsic infections are promoted by the patient’s gut flora (Dimitriu et al., 2019; Podder et al., 2019). While infectivity, pathogenicity, and virulence are the three main variables that determine the outcome in patients exposed to fungi, the course of the infection is also influenced by fungal cell wall (Rodrigues et al., 2007). The fungal cell wall, consisting of β-1,3-glucan, chitin, chitosan, and glycosylated proteins, mediates the innate immune response against fungal infection. Targeting the fungal cell wall has emerged as a promising strategy for antifungal drugs due to its specificity for fungi and the ability to induce cell lysis. Studies have shown that *Candida albicans* β-glucan can invade uninfected monocytes, exacerbating immunodeficiency and increasing susceptibility to systemic fungal infection (Nisini et al., 2007). Similarly, caspofungin has been found to enhance the immune system’s ability to destroy mold hyphae mediated by human polymorphonuclear neutrophils (Lamaris et al., 2008). Conversely, Maligie and Selitrennikoff (2005) found that *Cryptococcus neoformans* develop resistance to caspofungin and cilofungin through a mechanism unrelated to β-1,3-glucan synthase resistance. The structure of fungal cell wall and virulence factors are summarized in Figure 1.

Fungal pathogens have developed various strategies to evade immune recognition and cause systemic infections. Luo et al. demonstrated that *Candida albicans*, through sequence polymorphisms and differences in the expression levels of Gpm1 (phosphoglycerate mutase 1) and Pra1 (pH-regulated antigen 1), essential complement evasion proteins, increases its virulence by modulating immune fitness (Luo et al., 2015). Dasari et al. investigated the central immune evasion protein Aspf2 in *Aspergillus fumigatus* and found that it facilitates early infection stages by preventing host innate immune attacks and disrupting lung epithelial cell layers (Dasari et al., 2018). *Aspergillus fumigatus* also employs Dihydroxynaphthalene melanin (DHN-melanin) to inhibit acidification in the phagolysosome, interfering with the endocytic process and evading immune cell absorption (Thywissen et al., 2011; Akoumianaki et al., 2016). Furthermore, Taborda et al. discovered that the opsonic receptor CR3 plays a crucial role in antibody-mediated complement-independent phagocytosis of *Cryptococcus neoformans*, connecting the innate and adaptive immune systems (Taborda and Casadevall, 2002). These studies highlight the mechanisms by which fungal pathogens manipulate the immune response for their survival and dissemination.

Fungal pathogens employ various mechanisms to evade host defense and immune responses, including the detoxification of oxidative death mechanisms. *Candida albicans*, for example, utilizes the glyoxylate cycle to resist neutrophils and evade oxidative stress responses, enhancing its survival (Miramon et al., 2012). Despite their presence in the human microbiome, certain fungi can cause severe and fatal systemic infections, particularly in patients with haematologic malignancies like leukemia (Bhatt et al., 2011). Fungal dissemination into the bloodstream is a critical step in triggering invasive fungal infections (IFIs), leading to skin, bone, and central nervous system (CNS) involvement (Maphanga et al., 2020). *Cryptococcus neoformans*, by evading mucociliary clearance, enters deep alveolar spaces and subsequently targets the CNS after disseminating through the bloodstream (Rajasingham et al., 2017). The resistance of fungi to antifungal agents is also a concern, with specific mutations identified in *Candida albicans* that increase resistance without causing tissue damage or excessive inflammation. These mutations affect genes involved in cell signaling pathways, including those regulating pathogen recognition (Naseem et al., 2015; Wirnsberger et al., 2016; Xiao et al., 2016; Zhao et al., 2017).

### 2.2. Fungal EVs as virulence factors

**Figure 2:**
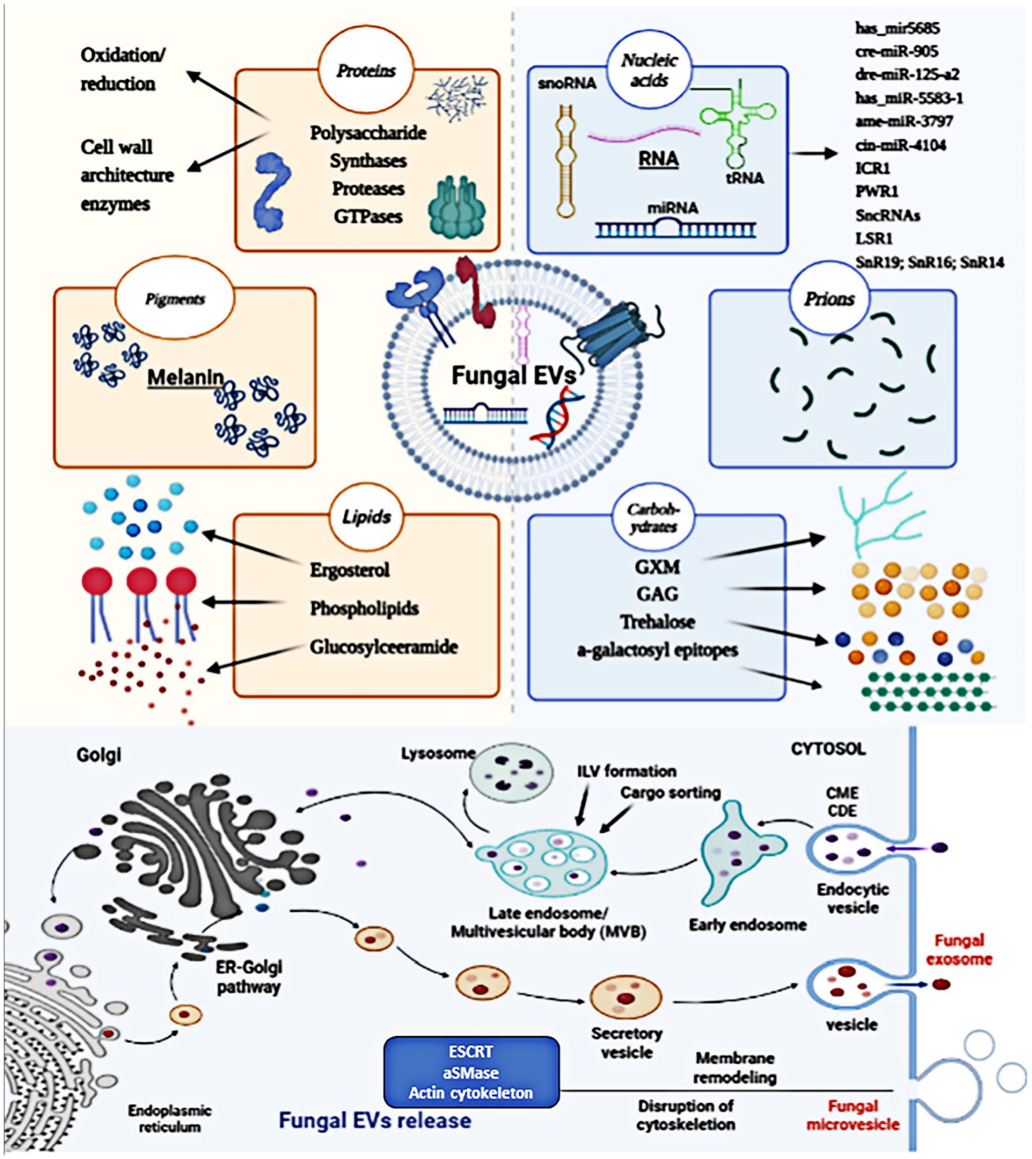
The structure and biogenesis of fungal EVs. Fungal EVs carry a variety of biologically native substances, such as nucleic acids, pigments, proteins, lipids, carbohydrates, polysaccharides, and prions, which are involved in pathophysiological processes. Fungal EVs biogenesis occurs in MVB endosomes, giving rise to secreted exosomes, and at the plasma membrane, resulting in the generation of MVs. Fungal microvesicle biogenesis occurs via the direct outward budding and pinching of the plasma membrane releasing the nascent microvesicle into the extracellular space.

The mechanisms of biogenesis and release of fungal EVs are not as well elucidated as in mammals. Genetic studies have implicated at least three different pathways in the release of EVs in fungi, including the conventional post-Golgi secretory pathway, ESCRT-mediated release of exosomes via MVBs, and an unconventional secretory pathway involving Golgi reassembly stacking proteins (GRASP). Fungal microvesicle biogenesis occurs via the direct outward budding and pinching of the plasma membrane releasing the nascent microvesicle into the extracellular space. Figure 2 summarizes the structure and generation of fungal EVs. Fungal EVs contain various native substances, including nucleic acids, pigments, proteins, lipids, carbohydrates, polysaccharides, and prions, which play roles in pathophysiological processes. Genetic and molecular evidence indicates similarities between fungal EVs and mammalian EVs (TerBush et al., 1996). Transmembrane proteins (annexins and tetraspanins), vesicle trafficking proteins (ESCRT proteins and Rab GTPases), cytoskeletal proteins, heat-shock proteins, metabolic enzymes, integrins, 14-3-3 proteins, and ribosomal subunits are all connected with mammalian EVs (Bonsergent et al., 2021). Similarly, fungal EVs were frequently abundant the same proteins involved in the plasma membrane, pathogenicity, stress responses, transport, signaling, and fundamental cellular metabolism (Zarnowski et al., 2021). Fungal EVs carry enzymes involved in metabolic pathway, such as amino acid biosynthesis and fatty acid metabolism, although their specific biological functions are yet to be fully understood (Rodrigues et al., 2007; Albuquerque et al., 2008; Vallejo et al., 2012b; Vargas et al., 2015). Comparable to EVs derived from tumors, fungal EV biogenesis and cargo loading involve the ESCRT-mediated MVB pathway and the conventional secretory pathway via the endoplasmic reticulum (ER)-Golgi Apparatus (GA)-exocyst-plasma membrane axis, with potential alternative pathways (Wenzel, 1995). Fungal EVs and mammalian EVs also show strong similarities in molecular content, such as both EVs transport several RNAs belonging to broad functional classes, including various functional classes of RNAs, such as messenger RNA (mRNA) (Lasser et al., 2017), which contribute to their biological functions. Table 1 summarizes early evidence and recent findings in the field of fungal EVs.

**Table 1:** The early evidence and recent discoveries in the field of fungal EVs.

Timeline	Discoverer	Fungal special	Discovery	References
1972	Gibson and Peberdy	*Aspergillus nidulans*	EVs release by *Aspergillus nidulans*	Gibson and Peberdy (1972)
1973	Takeo et al.	*Cryptococcus neoformans*	EVs secretion out of the cell membrane in *Cryptococcus neoformans*	Takeo et al. (1973)
1977	Chigaleichik et al.	*Candida tropicalis*	EVs term was used for the first time in *Candida tropicalis*	Chigaleichik et al. (1977)
1990	Anderson et al.	*Candida albicans*	EVs traversing the wall through “pimples” in *Candida albicans*	Anderson et al. (1990)
2007	Colmenares	*Candida albicans*	EVs released by Schizosaccharomyces pombe and *Candida albicans*	Colmenares et al. (2007)
2007	Rodrigues et al.	*Cryptococcus neoformans*	First description of fungal EVs in *Cryptococcus neoformans*	Rodrigues et al. (2007)
2008	Albuquerque et al.	*Histoplasma capsulatum; Candida albicans; Candida parapsilosis; Sporothrix schenckii and Saccharomyces cerevisiae*	EVs in *Histoplasma* spp.; *Candida* spp.; *Sporothrix* spp. and *Saccharomyces* spp.	Albuquerque et al. (2008)
	Rodrigues et al.	*Cryptococcus neoformans*	Virulence-associated proteins in *Cryptococcus neoformans*	Rodrigues et al. (2008a)
2009	Eisenman et al.	*Cryptococcus neoformans*	EVs in *Cryptococcus neoformans* melanization	Eisenman et al. (2009)
	Panepinto et al.	*Cryptococcus neoformans*	Sec6 is involved in EVs release in *Cryptococcus neoformans*	Panepinto et al. (2009)
2010	Oliveira et al.	*Saccharomyces cerevisiae*	Characterization of Saccharomyces spp. EVs and the effect of Sec4 in EVs release	Oliveira et al. (2010b)
		*Cryptococcus neoformans*	*Cryptococcus neoformans* EVs modulate macrophages	
2011	Gehrmann et al.	*Malassezia sympodialis*	EVs in *Malassezia sympodialis* and modulation of PBMC	Gehrmann et al. (2011)
2012	Vallejo et al.	*Paracoccidioides brasiliensis*	Lipidomics of *Paracoccidioides brasiliensis EVs*	Vallejo et al. (2012a)
	Huang et al.	*Cryptococcus neoformans*	*Cryptococcus neoformans* EVs and brain infection	Huang et al. (2012)
2014	Rizzo et al.	*Cryptococcus neoformans*	Apt1p and EVs-GXM secretion in *Cryptococcus neoformans*	Rizzo et al. (2014)
	Silva et al.	*Alternaria infectoria*	EVs in *Alternaria infectoria*	Silva et al. (2014)
2015	Kabani and Melki	*Saccharomyces cerevisiae*	EVs export prions in *Saccharomyces cerevisiae*	Kabani and Melki (2015)
	Gil-Bona et al.	*Candida albicans*	Proteomic analysis of EVs in *Candida albicans*	Gil-Bona et al. (2015)
	Vargas et al.	*Candida albicans*	Characterization and immunobiology of *Candida albicans* EVs	Vargas et al. (2015)
	Peres da Silva et al.	*Paracoccidioides brasiliensis and Paracoccidioides lutzii*	EVs derived from *Paracoccidioides brasiliensis* and *Paracoccidioides lutzii* export glycans	Peres da Silva et al. (2015b)
	Wolf et al.	*Candida albicans*	Lipid metabolism and EVs release in *Candida albicans*	Wolf et al. (2015)
2016	Matos Baltazar et al.	*Histoplasma capsulatum*	Humoral responses affect composition of Histoplasma capsulatum EVs	Matos Baltazar et al. (2016)
	da Silva et al.	*Paracoccidioides brasiliensis*	Paracoccidioides brasiliensis EVs modulate host innate immune response	da Silva et al. (2016)
2017	Rizzo et al.	*Cryptococcus neoformans*	*Cryptococcus neoformans* EVs modulate amoeba antifungal properties	Rizzo et al. (2017)
	Rayner et al.	*Malassezia sympodialis*	RNA export in Malassezia sympodialis EVs	Rayner et al. (2017)
2018	Leone et al.	*Pichia fermentans*	*Pichia fermentans* EVs in biofilm formation	Leone et al. (2018)
	Ikeda et al.	*Sporothrix brasiliensis*	EVs in Sporothrix brasiliensis and the modulation of dendritic cells and *in vivo* infection	Ikeda et al. (2018)
	Bitencourt et al.	*Trichophyton interdigitale*	EVs in Trichophyton interdigitale and modulation of macrophages and keratinocytes	Bitencourt et al. (2018)
	Peres da Silva et al.	*Cryptococcus neoformans*	EVs-RNA export and GRASP in *Cryptococcus neoformans*	Peres da Silva et al. (2018)
	Johansson et al.	*Malassezia sympodialis*	Allergens in Malassezia sympodialis EVs and interaction with skin cells	Johansson et al. (2018)
	Zarnowski et al.	*Candida albicans*	Biofilm EVs and ESCRT machinery in *Candida albicans*	Zarnowski et al. (2018)
	Liu et al.	*Rhizopus delemar*	EVs-exRNAs in Rhizopus delemar	Liu et al. (2018)
	Bielska et al.	*Cryptococcus gattii*	*Cryptococcus gattii* EVs and virulence transmission	Bielska et al. (2018)
2019	Souza et al.	*Aspergillus fumigatus*	EVs in *Aspergillus fumigatus* and interaction with macrophages and neutrophils	Souza et al. (2019)
	Reis et al.	*Cryptococcus gattii*	EVs-RNA and GXM export in Cryptococcus gattii. Novel protocol for fungal EVs isolation	Reis et al. (2019)
	De Paula et al.	*Trichoderma reesei*	EVs-associated cellulases in *Trichoderma reesei*	de Paula et al. (2019)
	Alves et al.	*Histoplasma capsulatum*	RNA export in Histoplasma capsulatum EVs	Alves et al. (2019)
	Zhao et al.	*Saccharomyces cerevisiae*	EVs and cell wall remodeling in *Saccharomyces cerevisiae*	Zhao et al. (2019)
2020	Winters et al.	*Saccharomyces cerevisiae*	IVC organelles and EVs secretion in *Saccharomyces cerevisiae*	Winters et al. (2020)
	Dawson et al.	*Candida albicans*	Claudin-like Sur7 family proteins as potential markers of *Candida albicans* EVs	Dawson et al. (2020)
	Bleackley et al.	*Fusarium* spp.	EVs in *Fusarium oxysporum* f.sp. vasinfectum and phytotoxicity	Bleackley et al. (2020)
	Vallhov et al.	*Malassezia sympodialis*	EVs in Malassezia sympodialis and interaction with human keratinocytes	Vallhov et al. (2020)
	Kabani	*Saccharomyces cerevisiae*	Glucose availability and EVs prions export in *Saccharomyces cerevisiae*	Kabani et al. (2020)
	Cleare et al.	*Histoplasma capsulatum*	Nutritional environment and EVs release in Histoplasma capsulatum	Cleare et al. (2020)
	Brauer et al.	*Aspergillus flavus*	Aspergillus flavus and their immunomodulatory functions	Brauer et al. (2020)
	Rizzo et al.	*Aspergillus fumigatus*	*Aspergillus fumigatus* EVs production in the absence of cell wall	Rizzo et al. (2020)
2021	Munhoz da Rocha et al.	*Candida auris*	generated data set for Candida auris and cellular small RNA fraction, EVss RNA during without/with caspofungin treatment	Munhoz da Rocha et al. (2021)
	Rizzo et al.	*Cryptococcus* spp.	Cryptococcus EVs has potential as a vaccine	Rizzo et al. (2021)
	Baltazar et al.	*Paracoccidioides brasiliensis*	Protective response in experimental Paracoccidioidomycosis elicited by EVs containing antigens of *Paracoccidioides brasiliensis*	Baltazar et al. (2021)
	Zamith-Miranda et al.	*Candida albicans; Candida auris*	Remarkably distinct compared to EVs from Candida auris’s phylogenetic relative *Candida albicans*	Zamith-Miranda et al. (2021)
	Yang et al.	*Talaromyces marneffei*	EVs derived from *Talaromyces marneffei* yeasts mediate inflammatory response in macrophage cells by bioactive protein components.	Yang et al. (2020)
2022	Castelli et al.	*Cryptococcus deuterogattii*	NOP16 functions in EV biogenesis and cargo, and the composition of EVs is determinant for cryptococcal virulence	Castelli et al. (2022)
	Amatuzzi et al.	*Candida auris*	The ability of Candida auris to efficiently alter the composition of EVs represent a mechanism for the fungus to mitigate the effects of antifungal agents	Amatuzzi et al. (2022)
	Gandhi and Joseph	*Candida albicans*	The potential use of EVs as theranostic marker for management of fungal infections	Gandhi and Joseph (2022)
	Las-Casas et al.	*Fonsecaea* spp.	Fonsecaea produce EVs capable of modulating pro- and anti-inflammatory cytokine and nitric oxide production by BMDMs and that growth conditions affected the immunomodulatory capacities of the EVs as well as their size, content, and morphology	Las-Casas et al. (2022)
	Martinez-Lopez et al.	*Candia albicans*	First analysis of HEVs of Candia albicans showed differences between them and the YEVs of Candia albicans	Martinez-Lopez et al. (2022)
	Kulig et al.	*Candida tropicalis, Candida glabrata, and Candida parapsilosis*	EVs not only in the physiology of *C. tropicalis*, *C. glabrata*, and *C. parapsilosis* fungi but also in the pathogenesis of infections associated with the production of fungal biofilm	Kulig et al. (2022)
	Octaviano et al.	*Paracoccidioides brasiliensis*	EVs from *Paracoccidioides brasiliensis* induce the expression of fungal virulence traits *in vitro* and enhance infection	Octaviano et al. (2022)
	Honorato et al.	*Candia albicans*	Candia albicans EVs inhibited biofilm formation *in vitro*	Honorato et al. (2022)
2023	Rizzo et al.	*Cryptococcus neoformans*	New mechanism of drug resistance in which cells adapt to azole stress by modulating EV production in *Cryptococcus neoformans* clinical isolates	Rizzo et al. (2023)
	Freitas et al.	*Aspergillus fumigatus*	*Aspergillus fumigatus* EVs play a role in protection against fungal infection	Freitas et al. (2023)

Many virulence factors are transported from fungal cells via EVs (Schrettl et al., 2007; Silva et al., 2014; Bleackley et al., 2020; Garcia-Ceron et al., 2021), including those that lack signal peptides (Liu et al., 2020). Studies have focused on profiling EVs-associated RNAs in fungi and host cells, investigating their accumulation during infection and identifying potential target genes. RNA content in EVs has been found to contribute significantly to gene regulation (Yanez-Mo et al., 2015). In invasive fungi like *Candida albicans*, *Aspergillus fumigatus*, and *Paracoccidioides brasiliensis*, different intraspecific cells exhibit varied gene regulation induced by EVs. Goncalves et al. identified 10 up-regulated lncRNAs, including 1200007C13Rik, 4833418N02Rik, Gm12840, Gm15832, Gm20186, Gm38037, Gm45774, Gm4610, Mir22hg, and Mirt1, associated with biological processes during *Candida albicans* infection, particularly the response to injury (Goncalves et al., 2023). In addition, Rosenblad et al. discovered three novel ncRNA genes, namely nuclear RNase P RNA, RNase MRP RNA, and a potential snoRNA U14, originating from the ribosomal region of fungi, highlighting the important role of ncRNA genes in fungal EVs genetics (Rosenblad et al., 2022). The emergence of an increasing number of fungi suggests that the diversity of fungal species is generally underestimated. EVs derived from different fungal species exhibit different virulence characteristics when infecting hosts.

Fungal EVs are considered crucial for microorganisms-host interactions. *Candida albicans*, a major cause of lethal fungal infections in immunocompromised individuals, employs adhesins, invasins, and hydrolases as virulence factors to breach physical barriers and invade host tissues (Saville et al., 2003). EVs have been successfully isolated from various *Candida* spp., including *Candida glabrata, Candida parapsilosis*, and *Candida tropicalis* (Wellington et al., 2009; Gil-Bona et al., 2015; Karkowska-Kuleta et al., 2020). The endosomal sorting complex required for transport (ESCRT) pathway, specifically subunits Hse1 and Vps27, is involved in *Candida albicans* biofilm EV production. Mutants deficient in ESCRT subunits show decreased EVs production (Zarnowski et al., 2018). Dawson et al. performed the novo identification of EV protein for *Candida albicans*, which found that claudin-like Sur7 family (Pfam: PF06687) proteins Sur7 and Evp1 (orf19.671) had the correlation with the fungal infection (Dawson et al., 2020). Additionally, differences in cytokine release were observed among Candida species, with *Candida glabrata* producing the highest levels (Kulig et al., 2022). Moreover, *Candida auris*, a chronic infectious disease, can be resistant to antifungal drugs. EVs purified from *Candida auris* improved antifungal resistance to amphotericin B formulations (Chan et al., 2022). The biological phases of *Candida albicans* are yeast and hyphae. Hyphae can damage tissue by infiltrating mucosal epithelial cells, which eventually causes a blood infection and hyphal EVs derived from *Candida albicans* differ from yeast vesicles due to containing a greater number of virulence-related proteins despite both kinds of EVs showed immune reactively with human sera from invasive candidiasis patients (Martinez-Lopez et al., 2022).

In addition to *Candida* spp., *Aspergillus fumigatus* also secretes EVs, leading to alterations in fungal cell wall morphology and exerting antifungal effects. This indicates the involvement of *Aspergillus fumigatus* EVs in inhibiting mycelial growth within the fungal cytoplasm, possibly due to the presence of an antimicrobial protein (Shopova et al., 2020). *Aspergillus fumigatus* possesses membrane-delimited organelles involved in melanization and is recruited to the endosome through an unconventional secretion pathway, utilizing various factors. This pathway allows for the sequential concentration and utilization of substrates, galactomannan-anchor precursors, anti-reactive oxygen species (ROS), and P450 (CYP450) enzymes (Upadhyay et al., 2016). Additionally, the endosomal system facilitates the export of negatively charged macromolecules across the plasma membrane, which helps sequester toxic intermediates and minimizes the risk of damage to other cytoplasmic machinery (Upadhyay et al., 2016). Souza et al. purified EVs from *Aspergillus fumigatus*, showing that the presence of glucanosyltransferases (Gel1, Gel4, and Bgt1), Ecm33, and EglC among proteins involved in cell wall remodeling in *Aspergillus fumigatus*, which resulted in the elongation of the cell wall glucan chain and resulting in the maintenance and resistance of the cell wall, indicating that these proteins in the EVs participate of mechanisms of fungal virulence (Souza et al., 2019).

Rodrigues et al. found that approximately 76 different proteins have been detected in *Cryptococcus neoformans* EVs and implied that *Cryptococcus neoformans* secretes pathogenesis-related chemicals in an effective and widespread manner, and that EVs serve as “virulence bags” that transport a concentrated payload of fungi to host effector cells and tissues. However, there is minimal overlap and no analysis of their abundance or enrichment (Rodrigues et al., 2008b). Among these proteins, the immunogenic properties of three *Cryptococcal* proteins (Cda2, Fpd1, and MP88) have been previously confirmed (Levitz et al., 2001; Biondo et al., 2003). Recent studies have identified key virulence factors in *Cryptococcus neoformans* EVs, including superoxide dismutase, phospholipase B, urease, and glucuronoxylomannan (GXM), a high-molecular-weight polysaccharide. Specifically, GXM is produced intracellularly and transported through the cell wall within these secretory vesicles (Mariano Andrade et al., 2003; Monari et al., 2005; Yauch et al., 2006; Rodrigues et al., 2007; Monari et al., 2009). *Cryptococcus* EVs play a crucial role in the pathophysiology of fungal infection processes by acting as virulence factors by regulating prion transmission, virulence transfer, and antifungal drug resistance. Furthermore, applying an innovative protocol for the new isolation of *Cryptococcus gattii* EVs, Reis et al. studied *Cryptococcus gattii* and revealed the involvement of scrablase a phospholipid translocase, as a virulence factor in *Cryptococcus gattii* secretion (Reis et al., 2019). EVs have also been observed in fungal culture supernatants and body fluids of various human pathogenic fungi, including *Histoplasma capsulatum* (Albuquerque et al., 2008), *Sporothrix brasiliensis* (Ikeda et al., 2018), *Sporothrix schenckii* (Albuquerque et al., 2008), *Malassezia sympodialis* (Gehrmann et al., 2011), and *Trichophyton interdigitale* (Bitencourt et al., 2018). Zhao et al. demonstrated that EVs enriched with *Saccharomyces cerevisiae* Fks1 and Chs3 protected yeast cells from cell wall disruption, suggesting that EVs prevent the host immune system from killing fungal organisms through a potential role in intraspecific fungal communication (Zhao et al., 2019). Rayner et al. reported that *Malassezia sympodialis* EVs exhibited an RNAi-independent route for biogenesis, but no evidence was found linking small RNA expression in *Malassezia sympodialis* EVs to pH changes (Rayner et al., 2017). Therefore, the role of virulence factors of *Malassezia sympodialis* EVs in the host remains unclear. Additionally, Bitencourt et al. identified specific miRNA sequences in *Paracoccidioides brasiliensis* (has-mir5685, cre-MIR905, dre-MIR-125-a2, and has-mir-5583-1) and *Saccharomyces cerevisiae* (ame-mir-3797 and cin-mir-4104), suggesting their potential virulence significance in fungal EVs (Peres da Silva et al., 2015b).

## 3. The interaction of fungal EVs with the immune system

**Figure 3:**
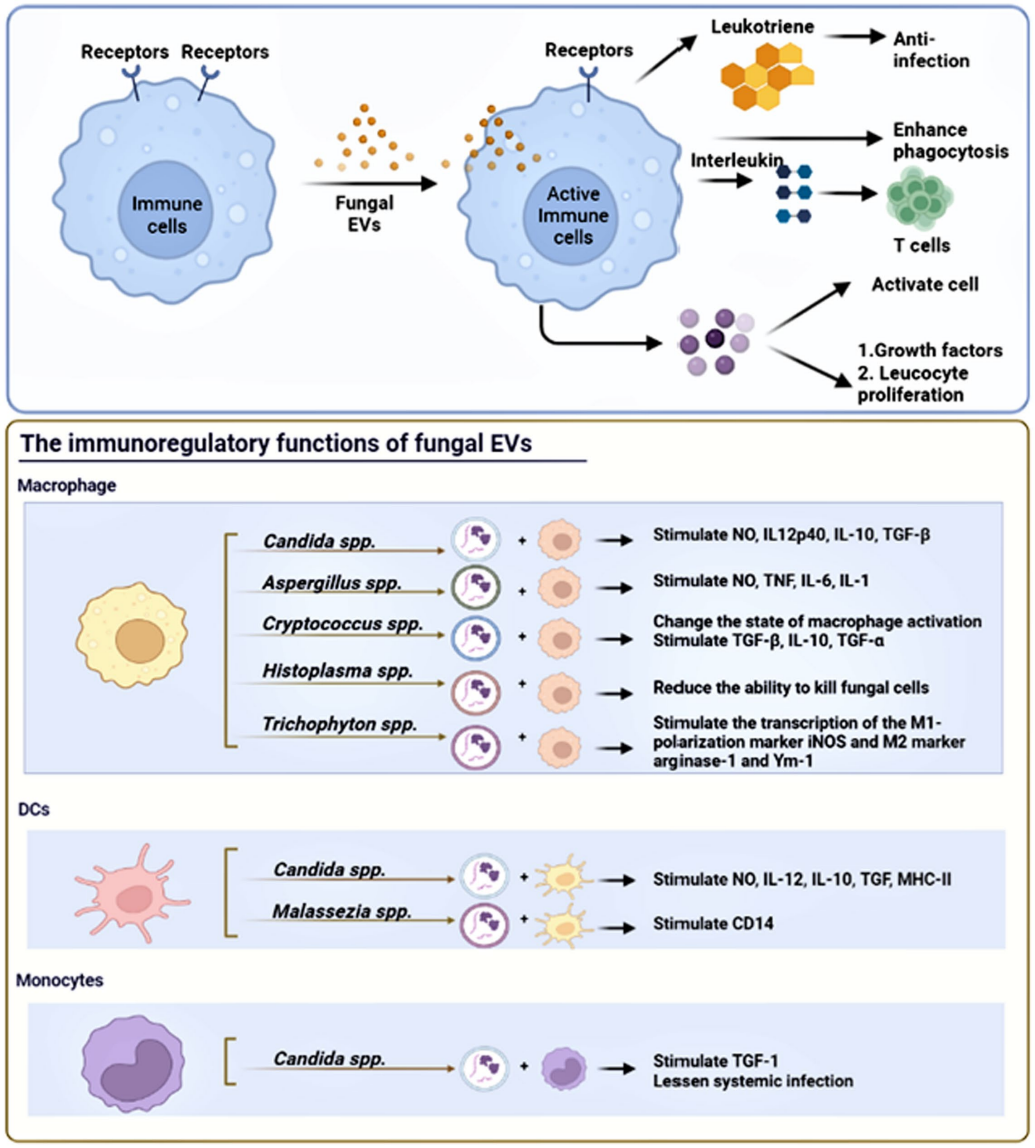
The immunoregulatory functions of fungal EVs. Fungal EVs can present antigen on their surface molecules directly to T cells, DCs, and monocytes. Fungal EVs could modulate positively or negatively the activation of innate immunity, which depend on the presence of fungal virulence factors and fungal species.

Fungi have developed effective immune evasion mechanisms to survive within the host environment, and long-term antigenic stimulation can significantly impact the modulation of the host immune response (Richie et al., 2009). Fungal pathogens possess the ability to evade human host immune responses by skillfully avoiding recognition and manipulating host responses, which may potentially trigger immune reactions against the fungi (Luo et al., 2015). Similarly, fungal EVs also have the capacity to modulate immunity activation, either positively or negatively, depending on the presence of fungal virulence factors (Oliveira et al., 2010a). Fungal EVs regulate the expression of co-stimulatory molecules in dendritic cells, macrophages polarization, and cytokine production in phagocytes. Figure 3 shows the immunoregulatory functions of fungal EVs. Table 2 summarizes the positive and negative regulation of activation of innate immunity by fungal EVs.

**Table 2:** Positively and negatively modulate the activation of innate immunity with fungal EVs.

Immune effects	Fungal species	Mechanisms	Outcomes	References
Positive regulation	*Candida albicans*	RAW264.7 macrophages produce NO, IL-12p40, and lower concentration of IL-10 and TGF-β; bone marrow-derived macrophages (BMDM) produce NO, IL-12p40, TNF-α, and TGF-β; via maturation, coincides with a higher expression of major histocompatibility complex class II and CD86	Activation of the innate response	Vargas et al. (2015)
	*Candida albicans*	*In vivo* study with larval	A lower fungal burden and an increased life span in the EV-treated larvae	Vargas et al. (2015)
	*Candida albicans*	EVs derived from phosphatidylserine synthase (CHO1)-knockout fungi were unable to activate NF-κB in BMDM and J774.14 macrophages	Immune cell activation	Wolf et al. (2015)
	*Cryptococcus neoformans*	Macrophages stimulated *in vitro* with EVs derived from *C. neoformans* produce anti-inflammatory cytokines, such as transforming growth factor β (TGF-β) and IL-10; produce TNF-α, NO	Anti-inflammatory function; increased ability to phagocytize and kill fungal cells	Oliveira et al. (2010a)
	*Paracoccidioides brasiliensis*	Induce the differentiation of M1 macrophages; produce proinflammatory cytokines and increase relative expression of iNOS gene	Cause macrophage phagocytosis and modulate the innate immune response	da Silva et al. (2016)
	*Paracoccidioides brasiliensis*	The interaction between EVs and DCs was dendritic cell-specific intercellular adhesion molecule-3-grabbing nonintegrin (DC-SIGN) dependent	Generation of immune response	Peres da Silva et al. (2015a)
	*Malassezia sympodialis*	Carry allergens recognized by IgE from AE patients; induce the production of IL-4 and TNF-α by peripheral blood mononuclear cells (PBMC)	Exert an indirect immune effect	Gehrmann et al. (2011)
	*Malassezia sympodialis*	DCs are able to phagocytize these fungal structures and produce their own EVs containing *M. sympodialis* antigens. Monocytes and keratinocytes could actively internalize *M. sympodialis* EVs	Exert an indirect immune effect	Johansson et al. (2018)
	*Trichophyton interdigitale*	Toll-like receptor 2 activation; produce NO, TNF-α, IL-6, and IL-1β in response to EVs. Increase iNOS relative expression; EV-treated macrophages differentiate into M1 macrophages, exhibiting a higher phagocytic and intracellular killing index than other macrophages when incubated with fungal conidia	Induce a strong inflammatory response in BMDM and keratinocytes	Bitencourt et al. (2018)
Negative regulation	*Candida albicans*	Hgt1p in EVs plays a canonical transmembrane role in glucose transport, binding to FH on the surface of the yeast cell wall serves an additional non-canonical role as a moonlighting protein	Interfering with host cells, enabling *Candida albicans* to evade the immune attack	Karkowska-Kuleta et al. (2020)
	*Cryptococcus neoformans*	Carry many virulence factors, including its major capsular antigen, glucuronoxylomannan (GXM), and laccase, the enzyme responsible for melanin production	Exert an immunosuppressive action over macrophages, monocytes, neutrophils, and T lymphocytes	Rodrigues et al. (2007); Rodrigues et al. (2008b); Vecchiarelli et al. (2000); Monari et al. (2003); Vecchiarelli et al. (1996); Monari et al. (2002)
	*Cryptococcus gattii*	Enter infected macrophages and impact on the rate of fungal intracellular proliferation within the phagosome	Modulate macrophage responses, ultimately facilitating the intracellular multiplication of less virulent strains without interfering with the phagocytosis rate	Bielska et al. (2018)
	*Histoplasma capsulatum*	EVs reduced the phagocytic rates and intracellular fungal killing by Bone marrow-derived macrophage (BMDM)	Suppressive in the infection	Baltazar et al. (2018)
	*Sporothrix brasiliensis*	Differential production of IL-12 p40, TNF-α, and IFN-γ compared with that by nontreated BMDC and higher levels of phagocytosis but not intracellular killing of *S. brasiliensis* yeast cells by pretreated BMDC than by nontreated BMDC	Impaired the initial immune response	Ikeda et al. (2018)

### 3.1. Fungal EVs positively modulate the activation of immunity

*Candida albicans* is more prone to cause diseases ranging from superficial mucosal to life-threatening systemic infections in patients with malignancy, leading to a variety of diseases, including mucosal infection, candidemia and disseminated candidiasis. Antimicrobial peptides and the complement system are two important, evolutionary conserved systems discovered as the first line of defense against bacteria in relation to the humoral response against fungi (Wenzel, 1995; Pfaller et al., 1998; Steele et al., 1999). *Candida albicans* EVs exhibit immunomodulatory effects that active the innate immune response. Co-stimulation of RAW 264.7 macrophages with EVs induces the production of NO, IL-12p40, IL-10, and TGF-β, similar to bone marrow-derived macrophages (BMDM) that produce NO, IL-12p40, TNF-α, and IL-10. In an *in vivo* experiment by Vargas et al., immunosuppressed mice vaccinated with EVs + Freund’s adjuvant (ADJ) showed higher levels of TNFα, IL-12p70, and IFN-γ in the blood. However, IL-12p70, TGFβ, IL-4, and IL-10 levels also increased without EVs + Freund’s adjuvant (ADJ) treatment (Vargas et al., 2020). Macrophages and DCs can internalize *Candida* spp. EVs, triggering a significant biological response characterized by increased NO, IL-12, IL-10, and TGF-β secretion, and MHC-II expression in DCs (Vargas et al., 2015). The morphological transition between yeast and hyphal forms is known to be linked to the virulence of *Candida* spp., affecting traits such as the ability to penetrate host tissues. Interactions on the cell surface and secreted proteins play crucial roles in the initial interaction between *Candida* spp. and the host (Hazen, 1989). *Candida albicans* EVs modulate the immune response by secreting TGF-1 transporter from human monocytes. It has been discovered that *Candida*-glucans activate the complement receptor 3 (CR3) on the plasma membrane of monocytes, leading to the production of TGF-1-transporting EVs. These EVs suppress the immune response in blood vessels, stimulate endothelial cells to produce TGF, and attenuate systemic infection (Mobius et al., 2002; Andriantsitohaina and Papon, 2020). Martinez-Lopez et al. observed that EVs secreted by hyphal cells (HEVs) of *Candida albicans* differ from yeast EVs (YEVs), with HEV exhibiting higher virulence and more pronounced effects on human immune cells (Martinez-Lopez et al., 2022). Oliveira et al. showed that low levels of *Candida haemulonii* var. EVs are not recognized by the traditional pathway of the oxidative burst produced by macrophages, which allow the transport of virulence factors via EVs that are not recognized by the host immune system and could act as regulators during infections caused by *Candida haemulonii var*. High EV concentrations and *Candida haemulonii var.,* however, triggered microbicidal responses of macrophages (Oliveira et al., 2023). Therefore, EVs were shown to contribute to fungal virulence, and these vesicles may be a source of antigens that activate host immune responses and can be used to develop new therapeutic targets.

It has been reported that human neutrophils produce antifungal EVs against *Aspergillus fumigatus* (Shopova et al., 2020). The release of EVs by the pathogenic *Aspergillus fumigatus* was first demonstrated by Souza et al., showing that *Aspergillus* EVs can induce phagocytosis, production of pro-inflammatory mediators, and phagocyte recognition, all of which contribute to fungal elimination (Souza et al., 2019). Brauer et al. evaluated *Aspergillus flavus* EV production and immunomodulatory effects and observed that the presence of EVs induces the production of inflammatory mediators such as NO, TNF, IL-6, and IL-1 are produced by macrophages. Furthermore, *Aspergillus flavus* EVs regulate macrophage phagocytosis, killing, and M1 macrophage polarization *in vitro* (Brauer et al., 2020). Mesenchymal stem cells (MSCs) play a role in the fungal immune response. Studies found that EVs secreted by MSCs possess tissue repair capabilities, inhibit inflammation, improve immune response, and promote angiogenesis (Gatti et al., 2011). In a clinical model of allergic airway inflammation, Cruz et al. discovered that MSC-secreted EVs attenuated the Th2/Th17-mediated inflammatory response, thereby alleviating airway hyper-responsiveness induced by *Aspergillus filaments* (Cruz et al., 2015). This suggests that MSC-EV could be a valuable target in vaccine formulations. Freitas et al. evaluated the pro- and anti-inflammatory effects of *Aspergillus fumigatus* EVs *in vitro*, revealing EVs derived from *Aspergillus fumigatus* induced a partial proinflammatory response in macrophages by increasing the production of TNF-α and gene expression of induced nitric oxide synthase and adhesion molecules (Freitas et al., 2023).

In *Cryptococcus* spp., studies have shown that *Cryptococcus neoformans* EVs are more beneficial than detrimental to the host (Oliveira et al., 2010a). Oliveira et al. observed that *Cryptococcus neoformans* secretes EVs during interactions with phagocytes *in vivo*, which can directly modulate host-pathogen interactions and influence the outcome of *Cryptococcus neoformans*-macrophage interactions by regulating macrophage activation states (Oliveira et al., 2010a). Indeed, *Cryptococcus neoformans* EVs have been shown to stimulate macrophages *in vitro*, inducing the production anti-inflammatory cytokines such as TGF-β and IL-10 (Oliveira et al., 2010a). Additionally, it is crucial to investigate the role of *Cryptococcus* spp. EVs in inducing immunity, particularly in infected immunocompromised hosts, where innate immune cells develop a memory-like response to repeated exposure to *Cryptococcus neoformans* (Devi et al., 1991; Hole et al., 2019). The presence of functional T cells is undoubtedly essential for effective defense against *Cryptococcus neoformans*. Moreover, Oliveira et al. found that incubation of murine macrophage with *Cryptococcus neoformans* EV led to a significant elevation in extracellular levels of TNF-α, IL-10, and TGF-β, promoting localized fungal infection (Oliveira et al., 2010a).

It has been reported that various fungal species secrete EVs that modulate the immune response of patients. Bitencourt et al. were the first to report that *Trichophyton interdigitale* secretes EVs, which stimulate the transcription of the M1-polarization marker inducible nitric oxide synthase (iNOS) and inhibit the expression of the M2 markers arginase-1 and Ym-1. These EVs also induce the production of pro-inflammatory mediators by bone marrow-derived macrophages (BMDMs) and keratinocytes in a dose-dependent manner (Bitencourt et al., 2018). Gehrmann et al. discovered that EVs derived from DCs co-cultured with *Malassezia sympodialis* can carry *Malassezia sympodialis* antigens and stimulate cytokine release in autologous CD14 and CD34-depleted PBMCs from patients. Interestingly, they also found for the first time that *Malassezia sympodialis* EVs contain antigens and allergens that induce cytokine responses in CD14 and CD34-depleted PBMCs from patients (Gehrmann et al., 2011). To date, *Paracoccidioides brasiliensis* EVs have been shown to stimulate M1 Macrophages *in vitro*, leading to the production of pro-inflammatory cytokines and increased relative expression of the iNOS gene (da Silva et al., 2016).

### 3.2. Fungal EVs negatively modulate the activation of immunity

As mentioned above, EVs derived from fungal species contain a diverse range of virulence factors, regulators, and highly immunogenic components that may directly contribute to disease development (Vargas et al., 2020). Therefore, the presence of multiple virulence factors in fungal EVs suggests that their potential harm to the host cannot be disregarded entirely. However, only a limited number of studies have reported on fungal EVs negatively modulating the activation of host immunity, such as *Sporothrix* spp. and *Histoplasma* spp. (Baltazar et al., 2018; Ikeda et al., 2018). In an *in vivo* study, Ikeda et al. observed a significant increase in fungal load in animals infected with *Sporothrix brasiliensis*. They found that the amount of fungal growth in the tissue increased as the number of fungal EVs injected into the mice increased, suggesting that fungal EVs facilitate the growth of fungus in the host (Ikeda et al., 2018). Similarly, Baltazar et al. demonstrated that the determination of the pathogen’s intracellular fate depends on the signaling pathways activated when macrophages and *Histoplasma capsulatum* interact. Importantly, they investigated that *Histoplasma capsulatum*-EV-treated macrophages had a reduced ability to kill fungal cells, particularly phagocytes treated with 7B6-EV. The levels of glutathione peroxidase (C0NI23) in EVs were reduced due to 6B7mAb treatment. Therefore, yeast cells may be better equipped to handle peroxides produced by phagocytes because glutathione peroxidase is less abundant in 6B7-EV (Baltazar et al., 2018). Additionally, in the extensively studies case of the opportunistic fungal pathogen *Cryptococcus neoformans*, EVs contain the capsular antigen GXM, which has been shown to exert cytotoxic effects on macrophages directly through the Fas/FasL pathway and can suppress monocytes, neutrophils, and T lymphocytes (Diamond et al., 1972; Villena et al., 2008). Other evidence suggests that *Cryptococcus neoformans* EVs enhance brain infection by facilitating the crossing of the blood-brain barrier and modulating the host’s ability to fight against infection through cytokines induction, providing a remarkable example of cross-kingdom communication (Huang et al., 2012). Therefore, the functional role of fungal EVs in the host immune system is complex, and further molecular biology experiments are needed in the future to explore the main regulatory pathways, as well as key cytokines and proteins.

## 4. The application of fungal EVs

Although current multiple methods for diagnosing fungal infections are applied including culture, microscopic examination, molecular biology, histopathology, and serological tests (Ness et al., 1989), they cannot specifically detect invasive fungal diseases and accurately identify fungal species in an efficient and inexpensive manner. The development of novel diagnostic methods for fungi can benefit from investments in identifying fungal pathogens through EVs-based strategies, as they offer distinct advantages in disease biomarkers (Ahn et al., 2018). EVs have been found to carry reliable markers for the diagnosis of infectious diseases, including viral and misfolded proteins, enabling the development of sensitive tests that can identify the infection and monitor its progression. Infectious EVs are particularly useful in this regard due to their ability to detect both active and dormant intracellular infections (Kruh-Garcia et al., 2014). However, the absence of specific protein markers, such as those found in mammalian EVs, which have allowed for sophisticated isolation and analysis techniques, has hindered in-depth research into fungal EVs. For example, ESCRT proteins like Vps23 (Tsg101) and Bro1 (ALIX), involved in the biogenesis of fungal EVs, are not present in the cargo of fungal EVs. Tetraspanin homologs have been discovered in numerous fungi, including the model yeast *Saccharomyces cerevisiae*. Interestingly, 22 putative *Candida albicans* EVs protein markers were identified, including the claudin-like Sur7 family (Pfam: PF06687) proteins Evp1 (orf19.6741) and Sur7 (Dawson et al., 2020), providing evidence that fungal EVs can be useful tools for elucidating the role of EVs derived from fungi. Furthermore, Martinez-Lopez et al. discovered distinct differences between *Candida albicans* hyphal cells EVs (HEVs) and *Candida albicans* yeast EVs (YEVs), suggesting their relevance and potential use in developing new diagnostic markers and therapeutic targets for *Candida albicans* infection (Martinez-Lopez et al., 2022). Moreover, a broad-omics approach proposed by Zysset et al. could enhance our knowledge in the fields of fungal MV biology, biogenesis, composition, and immunomodulatory capabilities, and facilitating the discovery of new molecules with diagnostic biomarker potential (Zysset et al., 1988). Fungal EVs containing RNA could serve as potential diagnostic biomarkers due to their involvement in spliceosome machinery and pre-mRNA processing during fungal adaptation to different niches (e.g., cre-MIR905, has-mir5685, dre-MIR-125-a2, and has-mir5583-1) (Peres da Silva et al., 2015b). Interestingly, has-mir5685 was also found to be significantly decreased in breast cancer, while has-mir5583-1 has a strong correlation with nasopharyngeal carcinoma (Wen et al., 2019), indicating the possibility of fungal infection in tumor patients through RNA delivery and communication in EVs. However, most current molecular tests lack validity, exhibit cross-reactivity, and are limited to the detection of a few species. Therefore, the exploration of fungal EVs as diagnostic biomarkers is still in its early stages.

RNA-based methods, such as host-induced gene silencing and artificial vesicle-protected RNA antifungal strategies, have been proposed as EV-based approaches for preventing infectious diseases (Cai et al., 2018). Currently, only a limited number of vaccine candidates targeting vulvovaginal candidiasis are under development, and there are no licensed antifungal vaccines available (Levy et al., 1989; Odds, 2003). The development of antifungal vaccines focusing on the fungal cell wall presents attractive targets. Clinical trials are underway for a vaccine composed of the recombinant N terminus of Als3p, a surface protein expressed by *Candida albicans* that facilitates host entry. This vaccine has demonstrated effectiveness in treating oropharyngeal and vaginal candidiasis (Schmidt et al., 2012). Additionally, preclinical studies in rodents have shown promise for the major secreted aspartic protease (Sap2) as a vaccine against vaginal candidiasis (Vecchiarelli et al., 2012). Fungal EVs elicit robust immune responses in animals both *in vitro* and *in vivo*, suggesting their potential application as mycosis vaccines. The antifungal properties of *Candida* EVs have been extensively investigated, with several studies demonstrating their ability to inhibit the growth of other fungal strains. For instance, Vargas et al. discovered that EVs from *Candida albicans* exhibit a protective effect against candidiasis by stimulating macrophages and dendritic cells to produce cytokines and express costimulatory molecules (Vargas et al., 2020). Gandhi et al. revealed that EVs derived from fungal-infected Retinal Pigment Epithelial (RPE) cells may contribute to the pathogenesis of endophthalmitis and activate immune signaling pathways, indicating the potential of EVs as a cargo for treating fungal infections (Gandhi and Joseph, 2022). In an *in vivo* study, Souza et al. found that *Aspergillus fumigatus* EVs rescued 50% of mice with *Aspergillus fumigatus*-induced lethal fungal pneumonia, suggesting their potential use as immunizing agents (Souza et al., 2022). Shopova et al. discovered a previously unknown mechanism by which neutrophils combat *Aspergillus fumigatus* infection, holding significant clinical implications for the development of new antifungal therapies (Shopova et al., 2020). Specifically, the discovery that neutrophils release EVs carrying antifungal substances to target fungi highlights the diagnostic and therapeutic potential of these vehicles for fungal infections.

Furthermore, conducting additional research on the molecular mechanisms underlying this interaction has the potential to uncover novel targets for drug development. Ultimately, comprehending the intricate interplay between host defenses and fungal pathogens is critical for enhancing the outcomes of patients affected with invasive fungal infections (Shopova et al., 2020). Considering the substantial global burden of the disease on high-risk populations, the development of a vaccine against Cryptococcosis is an urgent priority. Wang et al. demonstrated that in immunocompromised animals, particularly those lacking CD4+ T cells, heat-killed fbp1 cells (HK-fbp1) from *Cryptococcus neoformans* can confer protection against a challenge by the virulent parental strain, suggesting its potential as a therapeutic agent for treating invasive *Cryptococcus* infection (Wang et al., 2023). Additionally, Specht et al. investigated four Cryptococcal proteins (GP-Cda1, GP-Cda2, GP-Cda3, and GP-Sod1) and their potential role as candidate vaccines, highlighting their ability to protect mice from a lethal Cryptococcal challenge (Specht et al., 2017). The clinical application of fungal EVs is summarized in Table 3.

**Table 3:** The clinical application of fungal EVs.

Component	Source	Isolation method	Application	References
Claudin-like Sur7 family (Pfam: PF06687) proteins Evp1 (orf19.6741) and Sur7	*Candida albicans*	Ultracentrifugation	Fungal specific proteins for diagnosis	Dawson et al. (2020)
Hyphal cells EVs (HEVs)	*Candida albicans*	Ultracentrifugation	Diagnostic markers and therapeutic targets for *Candida albicans* infection	Martinez-Lopez et al. (2022)
cre-MIR905, has-mir5685, dre-MIR-125-a2, and has-mir-5,583-1	*Paracoccidioides brasiliensis; Cryptococcus neoformans*	Ultracentrifugation	Diagnosis biomarkers	Peres da Silva et al. (2015b); Bitencourt et al. (2018)
Fungal EVs	*Candida albicans*	Ultracentrifugation	Protective effect against candidiasis through elicit the production of cytokines and the expression of costimulatory molecules by macrophages and DCs	Vargas et al. (2015)
EVs derived from fungal-infected Retinal Pigment Epithelial (RPE) cells	*Aspergillus flavus* *Candida albicans*	Ultracentrifugation	Potential use of EVs as a cargo for the treatment of fungal infections	Gandhi and Joseph (2022)
Fungal EVs	*Aspergillus fumigatus*	Ultracentrifugation	Useful tool as immunizing agents	Souza et al. (2022)
Fungal EVs	*Aspergillus fumigatus*	Ultrafiltration	Potential role of new antifungal therapies and the potential approach as a diagnostic tool	Shopova et al. (2020)

## 5. Conclusion and perspective

Fungal infections are increasingly prevalent worldwide. However, unlike bacteria or viral infections, there is currently no available vaccine for fungal threats. In recent years, the incidence of fungal infections has risen, particularly among immunocompromised individuals such as organ transplant recipients, AIDS patients, and those undergoing chemotherapy. The identification of fungal EVs has provided a promising avenue for diagnosing and treating invasive fungal infections. Nonetheless, the field of fungal EV research is still in its early stages within mycology, despite the widespread use of EVs in various biomedical disciplines. The impact of fungal EVs on the host’s innate immunity can be either beneficial or detrimental, contingent upon the presence of fungal virulence factors. Interestingly, mounting evidence suggests that fungal EVs can serve as valuable tools for investigating the role of EVs derived from fungi. This review highlights the current progress and limitations in the study of fungal EVs, encompassing their potential clinical applications as diagnostic tools and therapeutic carriers. However, despite the growing interest and understanding of fungal EVs, their precise role in mediating the immune response between fungal cells and the host remains unknown. This includes elucidating the mechanisms by which fungal EVs enable fungal cells to evade immune surveillance and exploring the protective effects of fungal EVs in eliminating fungal pathogens. Furthermore, this review offers insights into the potential utility of fungal EVs in modulating host immune responses, improving antifungal drug delivery, and serving as a platform for vaccine development. It may also shed light on the unexplored therapeutic potential of fungal EVs in pathogenesis and their application in therapeutic interventions.

## Author contributions

JL was the major contributor in designing and writing the manuscript. XH provided substantial advice in designing and revising the paper, supervised the study, and contributed to manuscript preparation. All authors contributed to the article and approved the submitted version.

## Funding

This work was supported by grants from the Guangdong Basic and Applied Basic Research Foundation, Shenzhen Key Medical Discipline Construction Fund, Scientific Research Foundation of PEKING UNIVERSITY SHENZHEN HOSPITAL, Grant/Award Number: 2021A1515011558, SZXK040, and KYQD2021049.

## Conflict of interest

The authors declare that the research was conducted in the absence of any commercial or financial relationships that could be construed as a potential conflict of interest.

## Publisher’s note

All claims expressed in this article are solely those of the authors and do not necessarily represent those of their affiliated organizations, or those of the publisher, the editors and the reviewers. Any product that may be evaluated in this article, or claim that may be made by its manufacturer, is not guaranteed or endorsed by the publisher.

## Footnotes

^1^ http://www.gaffi.org/

^2^ http://www.who.int/mediacentre/factsheets/fs094/en/

^3^ http://www.who.int/mediacentre/factsheets/fs104/en/
